# Oriented Surface Immobilization of Antibodies Using Enzyme-Mediated Site-Specific Biotinylation for Enhanced Antigen-Binding Capacity

**DOI:** 10.1021/acs.langmuir.5c00656

**Published:** 2025-04-20

**Authors:** Emily Beitello, Kwame Osei, Trent Kobulnicky, Faith Breausche, Jon A. Friesen, Jeremy D. Driskell

**Affiliations:** Department of Chemistry, Illinois State University, Normal, Illinois 61790, United States

## Abstract

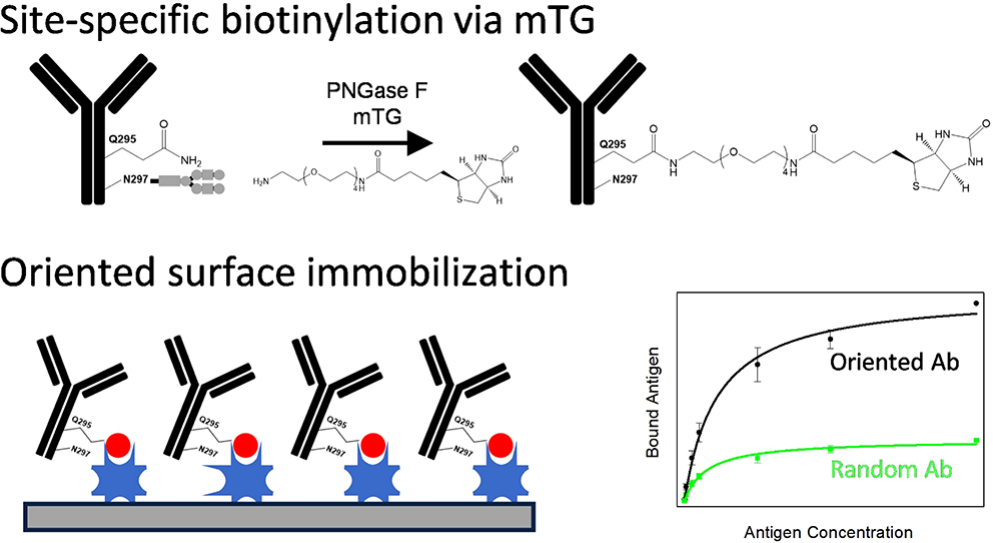

The effectiveness of surface-immobilized antibodies is often diminished by improper antibody orientation and limited stability, impeding the analytical performance of biosensors. Here, we report a novel enzyme-mediated strategy to biotinylate the Fc region of an anti-horseradish peroxidase (anti-HRP) antibody with site-specificity that enables oriented immobilization on a streptavidin-functionalized surface. Microbial transglutaminase (mTG) catalyzes the covalent coupling between the amine functional group on a biotin analogue (NH_2_-PEG_4_-biotin) and the side chain of a privileged glutamine residue (Q295) located on the heavy chain Fc region of IgG antibodies. For comparison, an anti-HRP antibody was biotinylated using an amine-reactive biotin analogue (NHS-PEG_4_-biotin) to covalently couple to lysine residues randomly located throughout the antibody. The antibody that reacted with a 40-fold excess of biotin reagent formed conjugates with a biotin-to-antibody ratio of 1.9 ± 0.3 and 5.0 ± 0.6 for the site-specific and random biotinylation strategies, respectively. Western blot analysis confirms that mTG-mediated biotinylation is restricted to the heavy chain, while lysine-targeted biotinylation is observed on both the heavy and light chains. The site-specific and randomly biotinylated antibodies were immobilized onto streptavidin-coated polystyrene 96-well plates to evaluate antigen (HRP) binding activity. The site-specific biotinylated antibody provided a 3-fold improvement in antigen binding capacity, sensitivity, and detection limit, that is attributed to the proper orientation of the antibody when immobilized through the Fc region. This chemo–enzymatic strategy is universally applicable to other antibodies for oriented antibody immobilization via site-specific linking chemistries without the need for protein engineering.

## Introduction

Immobilization of antibodies onto a solid surface is fundamental to the development of immunosensors. The antibodies are largely responsible for providing desirable analytical metrics, including high sensitivity and specificity; however, achieving optimal performance requires dense, robust, and oriented immobilization of the antibodies. Given its critical importance, numerous immobilization strategies have been explored in search of a method that delivers key immobilization criteria.^1,2^ A commonly utilized and simple approach to antibody immobilization is physical adsorption via hydrophilic interactions, e.g., polystyrene plates. While simple to implement, adsorption is protein-dependent, and the protein is susceptible to denaturation and desorption.^3−5^ It has been reported that as few as 10–20% of the physisorbed antibodies maintain an antigen-binding function.^6^ Leveraging the reactivity of amino acid side chains, such as amines and thiols, covalent coupling of the antibody to a surface can overcome challenges of desorption and stabilize protein structures to mitigate denaturation.^7−9^ In one quantitative study, use of covalent coupling to develop an immunosensor improved antibody function to ∼25% antigen-binding activity.^10,11^ Notably, both physical adsorption and covalent coupling result in a random orientation of the immobilized antibody molecules that causes the binding sites of many antibodies to be sterically blocked by the surface. Localized hydrophilic regions on the antibody are randomly distributed throughout the structure of the antibody to afford physical adsorption through many random points of contact. Likewise, lysine and cysteine residues are positioned throughout the antibody, and covalent attachment can occur through any of these linkages to generate a heterogeneous distribution of orientations. Consequently, the low density of functional antibodies available on an immunosensor developed with these immobilization techniques limits analytical performance.

Oriented immobilization of antibodies has the potential to enhance antigen-binding capacity and immunosensor sensitivity; thus, it is a critical issue for biosensor design.^1,2,12−14^ Several methods have been developed specifically to enable the orientation control of the immobilized antibody. To this end, surfaces are often first functionalized with protein A or protein G as an intermediate layer due to their specificity to bind the Fc region of antibodies, which then facilitates adsorption of antibodies in a preferred orientation.^12,13,15,16^ However, the binding affinity between the antibody and these Fc-binding proteins varies among antibody species and subtypes, and the reversible interaction can lead to loss of the antibody layer in subsequent assay steps.^13,17,18^ In another approach, whole IgG antibodies were treated with 2-MEA to reduce disulfide bridges, splitting the IgG antibody into two identical fragments. The fragmented antibodies were then directionally adsorbed onto a gold substrate via the newly generated thiols. This was effective in enhancing the performance of an immunosensor when directly compared to those relying on covalent coupling (e.g., random orientation) or protein G-mediated immobilization (e.g., oriented).^12^ However, this requires careful control over reducing conditions for reproducible fragmentation that varies with antibody subtype, and the fragments are prone to aggregation in solution.^19^

More recent work has focused on the development of novel ligands that selectively bind the Fc region of antibodies with high affinity. For example, a short peptide ligand was reported to mediate the oriented immobilization of a mouse IgG2a anti-prostate-specific antigen (anti-PSA) antibody to substantially improve the detection limit of an SPR-based sensor compared to the randomly immobilized antibody.^19^ However, much like the challenges associated with protein A and protein G, the selectivity and affinity of the peptide–antibody interaction are likely antibody-dependent. The instability and reversibility can be addressed with the incorporation of a noncanonical amino acid in the peptide ligand that reacts with the antibody to form a covalent bond when photoactivated;^20^ however, this requires expertise in protein engineering that is not universally accessible. Similar to Fc-binding peptides, aptamers have also been designed for oriented antibody immobilization.^21−23^ Compared to peptides, aptamers are easily and reproducibly synthesized and provide the opportunity to incorporate a photoinduced covalent cross-linking probe without the technical barrier associated with protein engineering.^24^

In contrast to a mediator-based approach, conjugation chemistry or affinity elements can be integrated into the antibody itself via protein engineering. Recombinantly expressed antibodies with a histidine tag incorporated into the molecular structure in the Fc region are one approach to achieve oriented immobilization.^25−27^ The his-tag spontaneously coordinates to metal ions, such as Ni^2+^ and Co^2+^, that are immobilized on a surface by nitrilotriacetic acid (NTA)-chelation chemistry. The biologically inspired SpyTag and SpyCatcher are peptide/protein binding partners that specifically and rapidly form an amide bond and have also been leveraged for oriented immobilization.^28,29^ This strategy requires integration of the SpyTag, a 13-amino acid sequence, into the antibody sequence via protein engineering for subsequent immobilization on a SpyCatcher-functionalized surface. These engineered affinity tags offer key features for optimal protein immobilization, such as controlled orientation, strong interaction, and spatially separated modification from the active binding site to maintain protein function. However, as previously noted, this approach is not amenable to widespread implementation due to the need for protein engineering.

Here, we developed a versatile approach to install a bioaffinity element at a site-specific location on an antibody without the need for protein engineering. Microbial transglutaminase (mTG) is a well-characterized enzyme that catalyzes the formation of an amide bond between a primary amine substrate and the γ-carboxamide group of a glutamine side chain.^30−33^ mTG has been found to be nondiscriminant toward the amine substrate; however, mTG recognition of the glutamine residue within a protein is more dependent on the local environment.^34^ Specifically, mTG has been reported to recognize a single glutamine residue, Q295, that is conserved in the Fc region of the IgG heavy chain.^35−39^ The site-specificity of mTG toward Q295 in human IgG antibodies has been exploited previously for the synthesis of antibody–drug conjugates with stoichiometric conjugation that does not interfere with antigen-binding function of the antibody. Leveraging this unique chemo–enzymatic function of mTG, an aminated biotin analogue was installed onto an antibody to facilitate oriented antibody immobilization. In this study, a rat anti-horseradish peroxidase (anti-HRP) monoclonal antibody was selected as a model antibody to investigate mTG-mediated, site-specific biotinylation. The anti-HRP antibody enables a straightforward approach to quantify antigen-binding capacity of the immobilized antibody and assess analytical figures of merit in a binding assay format.^11,40,41^ Moreover, the conjugated biotin is an established means through which to immobilize the antibody onto a streptavidin-functionalized surface with high affinity.^42^ These studies establish that mTG catalyzes the site-specific biotinylation of a rat antibody for oriented immobilization on a biosensor surface. Furthermore, this immobilization strategy resulted in a 3-fold enhancement in antigen-binding capacity and limit of detection compared with the immobilization of a randomly biotinylated antibody using NHS-biotin that conjugated to lysine residues. The analytical advantage achieved with this strategy is similar to that reported for other oriented antibody approaches;^11,12,43,44^ however, mTG-mediated immobilization offers a few key advantages. Most notably, an appropriate chemical linker can be installed on an IgG from any host species, without protein engineering, to offer antibody-independent immobilization, overcoming the significant variation in binding affinity inherent to IgG-binding proteins, such as protein A and protein G.

## Experimental Section

### Materials and Reagents

Rat anti-horseradish peroxidase monoclonal antibody was purchased from Bio X Cell (catalog #BE0088). Microbial transglutaminase (mTG) was purchased from Zedira (catalog #T300). A PNGase F deglycosylation kit was purchased from New England Biolabs (catalog #P0704L). Magne Protein G Beads were purchased from Promega. Amino-polyethylene glycol-biotin (NH_2_-PEG_4_-biotin) was purchased from BroadPharm. The following items were purchased from Thermo Fisher Scientific: EZ-Link NHS-PEG_4_ Biotinylation Kit, phosphate-buffered saline (PBS) packets, horseradish peroxidase (HRP) (catalog #31490), ammonium persulfate, glycine, tris(hydroxymethyl)aminomethane (Tris base), tris(hydroxymethyl)aminomethane hydrochloride (Tris HCl), methanol, potassium chloride, streptavidin-HRP (catalog #434323), 1-Step Ultra TMB-Blotting solution, PVDF/filter paper sandwich 0.2 μm pore size, streptavidin-coated clear 96-well plates, Immulon 2 HB clear 96-well plates, biotinylated recombinant protein G (catalog #29988), and 1-Step ABTS substrate solution. The following items were purchased from Sigma-Aldrich: Tween-20, bovine serum albumin (BSA) (catalog #A8806), sodium dodecyl sulfate (SDS), and acetic acid. Acrylamide/bis-acrylamide (37.5:1) was purchased from Research Products International. A Precision Plus Protein Dual Color Standards protein ladder was purchased from Bio-Rad. The following items were purchased from Nicoya: biotin–streptavidin sensor kit, flat-bottom polystyrene 96-well plates, and 10 mM glycine–HCl at pH 1.5.

### Site-Specific Biotinylation of Antibody with mTG

Prior to the site-specific biotinylation of the rat anti-HRP antibody using mTG, the IgG antibody was first deglycosylated. Following the manufacturer’s protocol, 10 μL of glycobuffer 2 (10×) was added to 100 μL of 1 mg/mL anti-HRP antibody in a microcentrifuge tube. PNGase F (2 μL) was added to the contents of the tube and mixed gently. The reaction mixture was then incubated at 37 °C overnight to allow for the deglycosylation of the antibody. mTG-mediated conjugation was immediately carried out with amino-PEG_4_-biotin after the deglycosylation reaction mixture cooled to room temperature. Briefly, 2.67 μL of 10 mM amino-PEG_4_-biotin solution was added to the tube to yield a 40-fold excess of biotin relative to the antibody. Next, 3.0 μL of 320 U/mL mTG was added for a final concentration of 8 U/mL, and the mixture was incubated at 37 °C for 24 h.

The antibody was isolated from the mixture containing excess biotin reagent, mTG, and PNGase F using Magne Protein G Beads. Following the manufacturer’s protocol, Magne Protein G Beads were equilibrated by pipetting 50 μL of the bead slurry into a 1.7 mL low-binding centrifuge tube. The tube was then placed in a magnetic stand for 10 s to separate the beads from the supernatant solution (storage buffer), and the storage buffer was removed and discarded. To reduce nonspecific binding, any vacant sites of beads were blocked using BSA. Separated beads were incubated for 15 min with 500 μL of 1% BSA prepared in PBS. After incubation, the tube was placed in the magnetic stand for 10 s, and the supernatant containing excess BSA was removed and discarded. The unpurified protein mixture (100 μL) was added to the blocked beads, and the mixture was incubated at room temperature for 60 min with intermittent gentle mixing. After incubation, the tube and its contents were placed in the magnetic stand for 10 s, and the supernatant containing unbound components (e.g., mTG, PNGase F, and excess biotin reagent) was removed and discarded. The antibody bound to the protein G beads was then washed twice with 500 μL of PBS, each incubating for 5 min with gentle mixing. As a final wash, the beads were gently mixed with 200 μL of PBS, and the supernatant was immediately discarded. The antibody bound to the beads was eluted by gently mixing the isolated beads with 40 μL of elution buffer (10 mM glycine–HCl, pH 2.7) for 5 min. The beads were magnetically separated from the suspension, and the supernatant (eluted antibody), approximately 40 μL, was immediately transferred to a new tube containing 8 μL of neutralization buffer (2 M Tris buffer, pH 7.5). The purified antibody was characterized for conjugation specificity and efficiency and used in a functional antigen-binding assay.

### Random Biotinylation of Antibody

Random biotinylation of the rat anti-HRP antibody was performed following the protocol of the Thermo Scientific EZ-Link NHS-PEG_4_ Biotinylation Kit. Preweighed 2.0 mg of NHS-PEG_4_-biotin was dissolved in 170 μL of water to create a 20 mM solution. To yield a 40-fold excess of biotin to antibody, 1.35 μL of NHS-PEG_4_-biotin was added to 100 μL of a 1 mg/mL anti-HRP antibody in a microcentrifuge tube. The reaction mixture was allowed to incubate at room temperature for 60 min. To remove the excess biotin linker from the solution, the biotinylated antibodies were purified using Magne Protein G Beads following the same procedure as the site-specific biotinylated antibodies.

### HABA–Avidin Quantitation of Biotin per Antibody

To quantify the amount of biotinylation on the rat anti-HRP antibodies, the HABA–avidin quantification procedure of the Thermo Scientific EZ-Link NHS-PEG_4_ biotinylation kit was followed. The HABA–avidin solution was prepared as 10 mg of avidin, 600 μL of 10 mM HABA, and 19.4 mL of PBS; when stored in the fridge, the quantification solution remained stable for 2 weeks. Biotinylation was quantified by the change in absorbance of the HABA–avidin solution before and after the introduction of the biotinylated sample. Biotin displaced the HABA molecule bound to avidin. Using a microvolume quartz cuvette, the absorbance of 90 μL of the HABA–avidin solution was measured at 500 nm. Next, 10 μL of purified biotinylated antibody (site-specific or random) was added directly to the HABA–avidin solution in the cuvette and gently mixed by a pipet. The absorbance of the solution was remeasured at 500 nm. Based on the concentration of the biotinylated antibody and the difference in absorbances, the number of biotin molecules per antibody was calculated.

### SDS-PAGE and Western Blot

Western blots were performed to verify the random and site-specific biotinylation of the antibody. Antibodies were first electrophoresed on a 12% SDS-PAGE gel under reducing conditions. A native antibody, site-specific biotin–antibody conjugate, and random biotin–antibody conjugate were combined with a DTT loading buffer in a microcentrifuge tube at a 3:1 (v/v) ratio of antibody to reductant and incubated at room temperature for 30 min with periodic mixing. After incubation, reduced samples were heated in a hot water bath (95 °C) for 5 min prior to being loaded in the gel. The SDS-PAGE gel was electrophoresed at 150 V for approximately 1.5 h or until the loading buffer dye reached the bottom of the resolving gel. Gels not used for a Western blot were stained overnight with a Coomassie blue solution (45% methanol, 10% acetic acid, and 0.1% Coomassie blue); then, to remove background staining, the gel was destained overnight (45% methanol and 10% acetic acid). In preparation for the Western blot transfer, the SDS-PAGE gel was soaked in a transfer buffer (24 mM Tris base, 192 mM glycine, and 20% methanol), and the PVDF membrane was soaked in methanol for 10 min. The membrane sandwich was then assembled as a sponge pad, filter paper, SDS-PAGE gel, PVDF transfer membrane, second filter paper, and second sponge pad. The membrane sandwich and an ice block, to prevent overheating during electrophoresis, were fully submerged in a tank of transfer buffer. With constant stirring, the system was electrophoresed at 100 V for 1 h. After the proteins were transferred from the gel to the PVDF membrane, the remaining empty space of the membrane was blocked with 10% nonfat dry milk in tris-buffered saline, pH 7.4 (100 mM Tris base, 137 mM NaCl, and 2.7 mM KCl). Blocking was incubated on a rocker for 1 h. After blocking, the membrane was washed three times, each 5 min on the rocker, by transferring the membrane to a clean container and covering it with approximately 25 mL of TBS-T (tris-buffered saline with 0.1% Tween-20). Next, the membrane was incubated with a streptavidin–horseradish peroxidase (SA–HRP) conjugate that will bind to any biotin molecules attached to the antibody fragments. SA–HRP was diluted 1:20,000 with TBS-T + 1% nonfat dry milk. Binding occurred on the rocker for 1 h. To remove any unbound SA–HRP from the surface, the membrane was thoroughly washed. Each wash consisted of approximately 25 mL of solution, and the membrane was incubated on a rocker for 5 min. First, the membrane was triple-washed with TBS-T, then triple-washed with TBS, and finally with a single wash of DI water. For detection, the membrane was submerged in 1-Step Ultra TMB-Blotting solution to generate a solid blue precipitate where SA–HRP bound to the PVDF membrane. Color development was closely monitored, and the reaction was stopped with two quick washes with DI water.

### SPR Characterization of Antibody–Antigen Binding

Surface plasmon resonance (SPR) analysis was conducted with an OpenSPR XT and a biotin–streptavidin sensor kit to assess the binding rate constants, *k*_on_ and *k*_off_, and the equilibrium dissociation constant, *K*_d_, of both random and site-specific biotinylated rat anti-HRP antibodies. The biotin sensor first underwent a surface cleaning step by injecting 10 mM glycine–HCl pH 1.5 at a flow rate of 150 μL/min for a total contact time of 40 s followed by treatment with PBS-T for 4.5 min. The sensor was then prepared with 0.5 μM streptavidin in PBS-T at a flow rate of 20 μL/min for a total contact time of 5 min followed by a PBS-T buffer rinse for 4.5 min. After functionalizing the surface with the streptavidin linker, the ligand, a random or site-specific biotinylated rat anti-HRP antibody, was immobilized onto the sensor surface. The purified antibody was diluted to 100 nM with PBS-T and then injected at a rate of 20 μL/min for a total contact time of 5 min followed by a PBS-T buffer rinse for 10 min. Antigen binding to the immobilized ligand was assessed by subjecting the sensor chip to increasing concentrations of HRP (4.9, 14, 44, 133, and 400 nM) prepared in PBS-T flowing at a rate of 20 μL/min for a total contact time of 5 min followed by a 10 min rinse with PBS-T. Between each injection of HRP, the sensor was regenerated by removing the bound HRP from the antibody with an injection of 10 mM glycine–HCl pH 1.5 at a flow rate of 150 μL/min for a total contact time of 40 s followed by a 4.5 min PBS-T rinse. All SPR data were exported to the TraceDrawer software and fit to a kinetic evaluation 1:1 binding model to extract the *k*_on_, *k*_off_, and *K*_d_ values.

### Antigen Capture Assay

Streptavidin-coated 96-well plates were utilized to assess antigen capture by the biotinylated anti-HRP antibodies. To determine how much antibody was required to saturate the surface of the wells, varying amounts of antibody were tested. The desired wells were first triple-washed with 150 μL aliquots of freshly prepared PBS-T (0.05% Tween-20). Antibody samples of 100 μL were added to duplicate wells at concentrations of 0, 1, 5, 10, 50, 100, and 200 nM. The well plate was covered with parafilm and allowed to incubate overnight at 4 °C. After incubation, the excess and unbound antibody was removed by triple-washing wells with 150 μL aliquots of PBS-T. The wells were then incubated with 100 μL of 1000 nM HRP, covered with parafilm, and incubated at room temperature for 30 min. Again, the wells were triple-washed with 150 μL aliquots of PBS-T to remove excess and unbound HRP. Then, to assess antigen capture, fully incubated wells were flooded with 150 μL of 1-Step ABTS solution, and the rate of HRP-catalyzed ABTS oxidation was spectrophotometrically measured at 410 nm with a plate reader (Thermo Varioskan LUX). Sufficient saturation of the streptavidin-coated wells was observed at a 100 nM biotinylated anti-HRP antibody. This antibody concentration was used to fabricate capture substrates for a functional assay to test the detection of variable antigen concentrations. For this capture assay, the streptavidin-coated wells were initially triple-washed with 150 μL of PBS-T and then incubated overnight with 100 μL of 100 nM biotinylated antibody at 4 °C covered. After incubation, the wells were triple-washed with 150 μL of PBS-T. For variable antigen concentrations, duplicate wells were exposed to HRP concentrations of 0, 0.1, 1, 5, 10, 50, and 100 nM. Antigen incubated in the capture well was covered at room temperature for 30 min. Sample wells were triple-washed with 150 μL of PBS-T and then flooded with 150 μL of ABTS to quantify antigen capture as it correlates to the rate of HRP-catalyzed ABTS oxidation.

## Results and Discussion

### Biotinylation and Characterization of Anti-HRP Antibody

**Figure 1:**
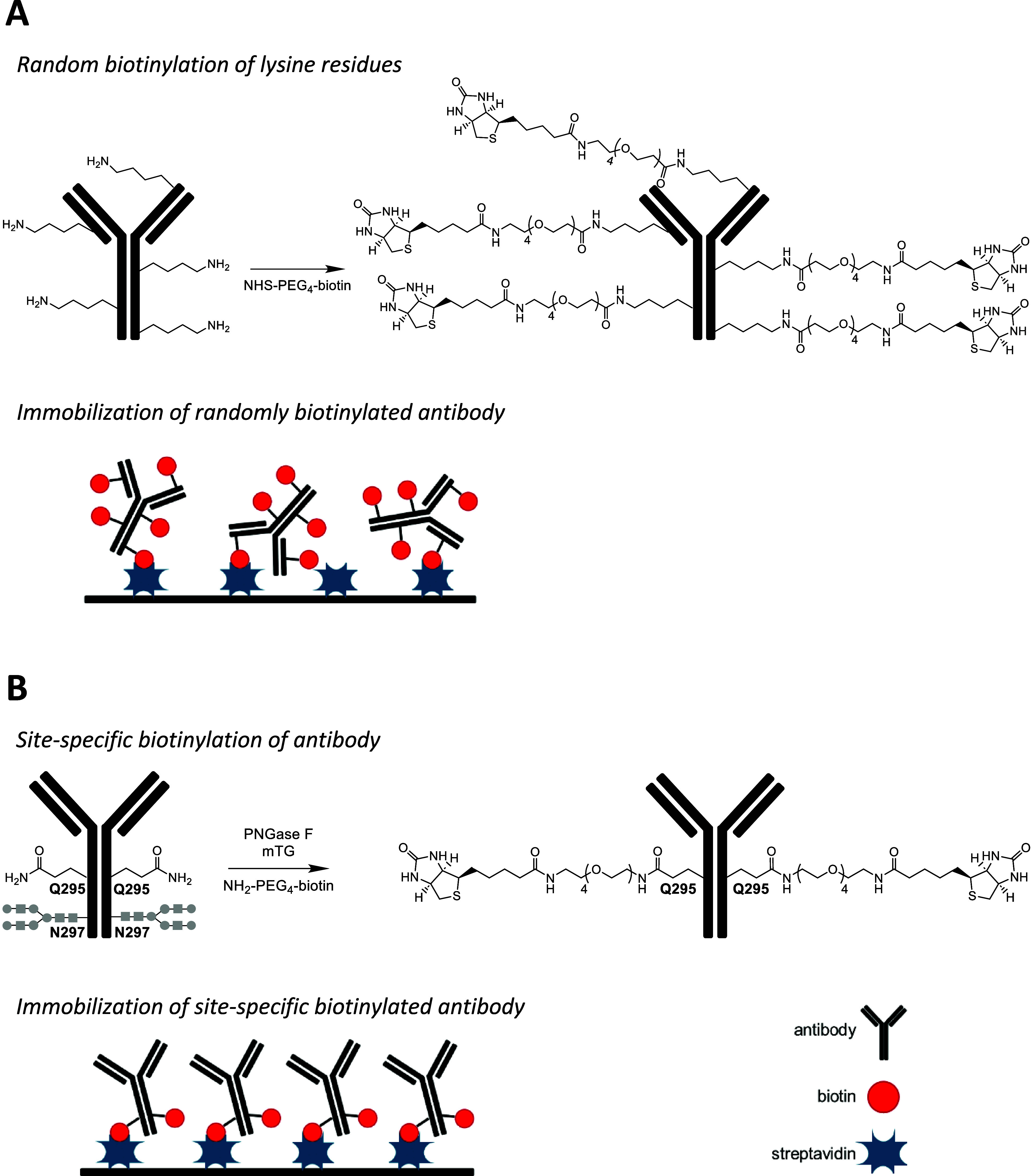
Random biotinylation reaction of the rat anti-HRP antibody with NHS-PEG_4_-biotin and an immobilization scheme on a streptavidin-coated surface (A). Site-specific biotinylation of the rat anti-HRP antibody with amino-PEG_4_-biotin mediated by mTG and an immobilization scheme on a streptavidin-coated surface (B).

Immobilization of antibodies on surfaces is central to the development of many bioanalytical assays and immunosensors. For example, antibodies are adsorbed onto a solid-phase surface to form an antigen capture substrate in the widely employed ELISA. Additionally, development of emerging technologies based on a sandwich immunoassay format often requires immobilization of capture antibodies on a solid-phase substrate and detection antibodies on the surface of nanoparticle-based labels.^45−48^ The immobilization strategy influences the surface density, orientation, and stability of the antibody, all factors that substantially impact the analytical performance of the functional assay.^1,2^ In this work, we exploit the robust biotin–streptavidin bioaffinity for antibody immobilization.^42^ Antibodies are readily biotinylated through covalent coupling of a biotin analog consisting of an activated ester (e.g., NHS) with reactive amines presented by lysine residue side chains.^7,8^ While effective, this results in biotinylating at random locations on the IgG molecule and ultimately random orientations upon immobilization on a streptavidin-coated surface (Figure 1A). Alternatively, we explored the use of microbial transglutaminase (mTG) as a strategy to site-specifically biotinylate the IgG molecule on the Fc region, thereby facilitating antibody immobilization on a streptavidin-coated substrate with a preferred orientation to enhance the antigen-binding capacity and analytical performance of immunosensors (Figure 1B).

mTG-mediated conjugation to the privileged Q295 site of IgG has been largely limited to human antibodies.^35−37^ In those previous works, it was established that deglycosylation of the antibody at N297 was necessary to eliminate steric hindrance to the nearby Q295 and enable sufficient conjugation efficiency.^37^ Thus, it is imperative to demonstrate the ability of mTG to facilitate conjugation of a rat IgG and investigate the need for deglycosylation. Following these previous studies, a monoclonal rat anti-HRP antibody was first treated with PNGase F to generate a deglycosylated antibody. A 40-fold excess of amino-PEG_4_-biotin and mTG was then added to either the deglycosylated antibody or native antibody (e.g., control IgG) to synthesize a site-specific biotinylated antibody. A randomly biotinylated antibody was also prepared as an additional control sample using a conventional conjugation strategy by reacting a 40-fold excess of NHS-PEG_4_-biotin with either the deglycosylated antibody or native antibody. The four biotinylated samples were then purified using protein G magnetic beads to isolate the antibody and remove excess reagents prior to characterization.

**Figure 2:**
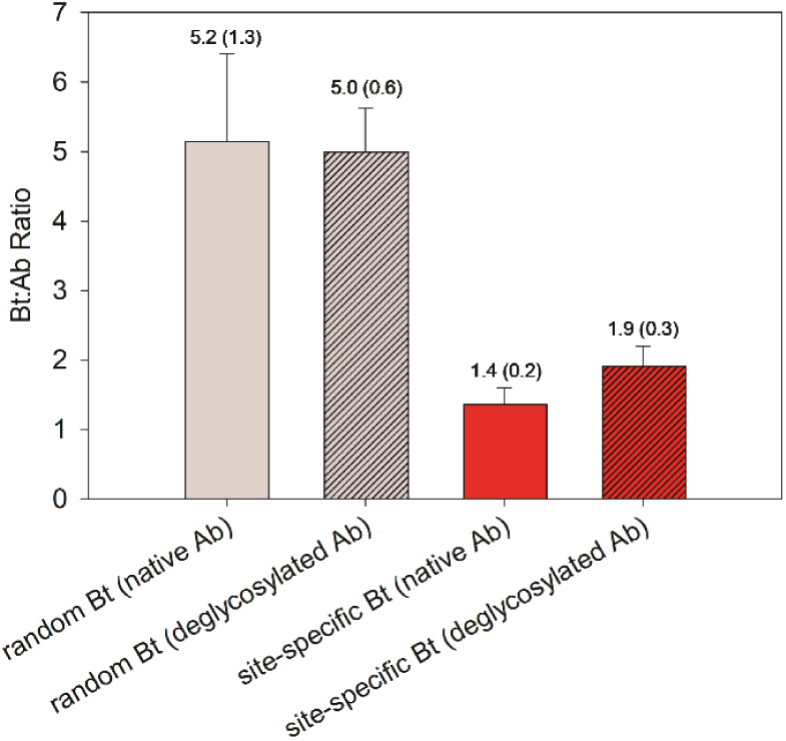
Quantity of biotin elements installed per antibody was determined by a HABA/avidin assay. All samples were reacted with 40-fold excess of biotin reagent and purified by protein G affinity magnetic beads. Each sample was independently prepared in triplicate. The bars represent the average values, and the error bars represent the standard deviations.

IgG molecules consist of two identical light chains and two identical heavy chains bound by disulfide bonds. mTG specifically catalyzes the reaction between a primary amine substrate and Q295 located on the heavy chain. Therefore, it is expected that the mTG-mediated, site-specific biotinylation strategy would result in the conjugation of two biotin molecules per antibody molecule, assuming ideal reaction efficiency and specificity. The quantity of biotin moieties conjugated to each IgG molecule was experimentally determined by using a HABA-based biotin quantitation assay. To this end, an avidin–HABA complex with a strong absorption band at 500 nm was mixed with the biotinylated antibody. Avidin preferentially binds to the conjugated biotin, releasing the avidin-complexed HABA to produce a nonabsorbing species. The difference in absorption of the avidin–HABA solution before and after the addition of biotinylated antibody correlates to the number of biotin units and is used to calculate the biotin:antibody mole ratio (Figure S1). The results of the biotin quantitation assay are presented in Figure 2. Site-specific biotinylation of the native antibody resulted in the conjugation of 1.4 ± 0.2 biotin units per antibody. Notably, deglycosylation improved the conjugation efficiency of the mTG-mediated biotinylation, resulting in the conjugation of 1.9 ± 0.3 biotin units per antibody. This suggests 95% conjugation efficiency based on a theoretical quantitative yield of 2 biotin groups per IgG and is consistent with improvements in mTG-mediated conjugation of IgG after deglycosylation. Random biotinylation of the native and deglycosylated antibodies at accessible lysine residues resulted in 5.2 ± 1.3 and 5.0 ± 0.6 biotin units per antibody, respectively. These results suggest that glycans on the antibody do not inhibit reactivity of available lysine residues, and this random approach achieves greater biotinylation of the antibody than mTG-directed conjugation.

**Figure 3:**
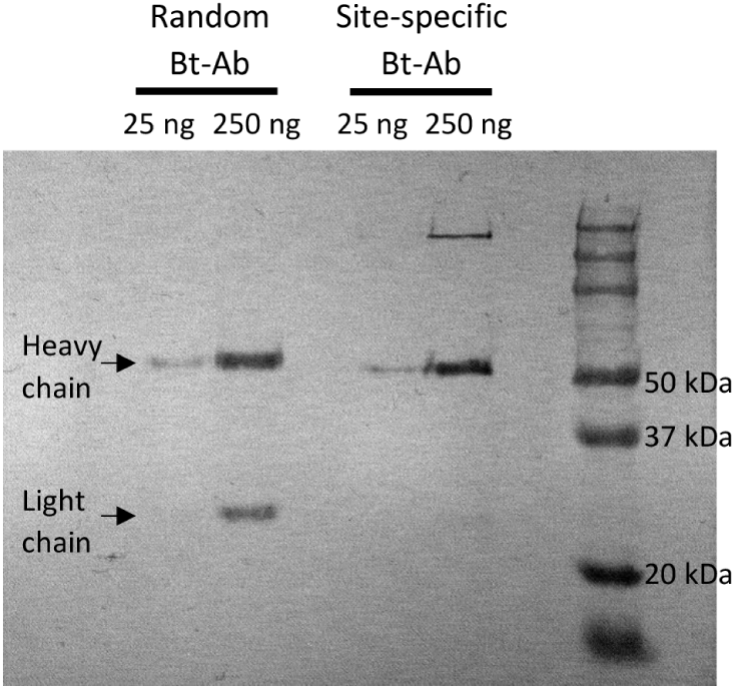
Western blot analysis of the reduced site-specific and random biotinylated antibody. The membrane was stained using HRP-labeled streptavidin and developed with TMB to visualize the antibody fragments with biotin moieties. Two masses of each antibody (25 and 250 ng) were loaded into the gel. Lanes (left to right): random biotinylated antibody (25 ng), random biotinylated antibody (250 ng), empty, site-specific biotinylated antibody (25 ng), site-specific biotinylated antibody (250 ng), empty, and molecular weight ladder.

A Western blot was performed to further characterize the biotinylated antibodies and confirm the specificity of mTG biotinylation to the IgG heavy chain. The randomly and site-specifically biotinylated antibodies were reduced and separated via SDS-PAGE before being transferred to a PVDF membrane. The transferred protein fragments were treated with HRP-labeled streptavidin to visualize only the biotinylated fragments (Figure 3). Biotin was incorporated into both the heavy (e.g., 50 kDa) and light (e.g., 25 kDa) chains for the antibody that reacted with NHS-PEG_4_-biotin. Presumably, this can be attributed to random reactivity with the numerous lysine residues located on both the heavy and light chains. In contrast, biotin was only installed onto the heavy chain of the antibody when the reaction with amino-PEG_4_-biotin was facilitated by mTG. While the light chain of the reduced site-specific biotinylated antibody was not observed in the Western blot, its presence was confirmed by SDS-PAGE gel stained with Coomassie blue (Figure S2). As noted, mTG is conventionally used to limit conjugation to Q295 of the human IgG heavy chain and has not previously been employed for rat IgG. BLAST sequence alignment indicated that Q295 is conserved in rat IgG, analogous to human IgG (Figure S3), and supports the experimental results presented here, demonstrating site-specific and efficient conjugation of two biotin units per rat IgG molecule.

Enzyme-directed biotinylation of the antibody does not impact antigen binding, given that the conjugation occurs at the Q295 site; however, it is possible that the antigen-binding parameters of the randomly biotinylated antibody could be adversely affected if a biotin was conjugated at or near the paratope. The binding rate constants, *k*_on_ and *k*_off_, and the equilibrium dissociation constant, *K*_d_, between the biotinylated antibodies and antigen (e.g., HRP) were quantitatively characterized by using surface plasmon resonance (SPR). The biotinylated antibodies were immobilized on a streptavidin-functionalized SPR sensor, and antigen solutions of varying concentrations were passed over the sensor to monitor association, followed by buffer to measure dissociation (Figure S4). The antibody binding metrics extracted from the best fit of the sensograms are listed in Table 1. Both biotinylated antibodies exhibited similar high affinities for HRP and similar binding kinetics, suggesting that the biotins randomly conjugated to the antibody were located a sufficient distance from the binding site such that it maintains the function of the native antibody. These results are key to establish that any differential analytical performance of an immunoassay developed with the site-specific biotinylated versus random biotinylated antibody can be attributed to differences in antibody immobilization rather than differences in antibody binding characteristics.

**Table 1:** Kinetics and Equilibrium Constants for the Binding of HRP to the Biotinylated Antibody

	*k*_on_ (M^–1^ s^–1^)	*k*_off_ (s^–1^)	K_d_ (M)
**random biotinylation**	1.25 × 10^5^	5.66 × 10^–4^	4.5 × 10^–9^
site-specific biotinylation	1.45 × 10^5^	4.93 × 10^–4^	3.4 × 10^–9^

### Antibody Immobilization and Antigen Capture in a Streptavidin-Coated 96-Well Plate

We selected a streptavidin-coated 96-well plate to evaluate the antigen-binding capacity of the randomly and site-specifically biotinylated antibodies. This is a commercially available solid-phase substrate that is commonly used as the foundational component in the development of an immunoassay. The choice of biotinylating an anti-HRP antibody in this work is convenient because it enables the facile assessment of antigen-binding capacity of the immobilized antibody by a colorimetric enzyme assay. More specifically, the immobilized anti-HRP antibody in the 96-well plate selectively captures HRP from the sample solution. Subsequently, ABTS is added to each well, and the number of captured HRP molecules correlates with the rate of HRP-catalyzed oxidation of ABTS to form a colored product.^11^

**Figure 4:**
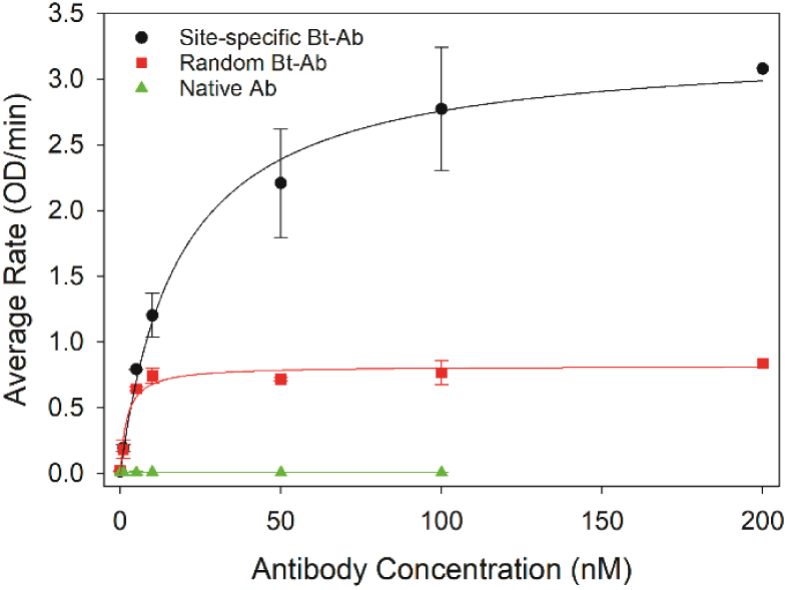
Dependence of antigen binding as a function of the antibody concentration. The site-specific biotinylated, random biotinylated, and native (nonbiotinylated) anti-HRP antibody was immobilized on a streptavidin-coated 96-well plate. Binding sites of the immobilized antibodies were saturated with excess HRP (1000 nM), and the rate of HRP-catalyzed ABTS oxidation was measured to quantify bound HRP. Data points and error bars represent the average values and standard deviations determined from three independent experiments, except for 200 nM (*N* = 1 or 0) that was included to confirm saturation.

It was necessary to determine the optimal concentration of the biotinylated antibody to immobilize on the streptavidin-coated wells as a first step toward the development of a functional bioassay. Ideally, the antibody concentration is optimized to maximize HRP capture without overcrowding that can lead to lower antigen binding.^49−51^ Random and site-specific biotinylated antibody solutions ranging from 1–200 nM were added to each well and allowed to adsorb. As a control, the streptavidin plate was also treated with the native antibody (e.g., nonbiotinylated) to assess nonspecific binding of antibody to the streptavidin layer. HRP was then added to each well at 1000 nM to ensure the saturation of all available binding sites presented by the immobilized antibodies. After removing the unbound HRP and thoroughly rinsing the well, ABTS was added to each well, and the rate of ABTS oxidation was measured spectrophotometrically (Figures 4 and S5). It has previously been established that the ABTS oxidation rate is directly correlated to the number of HRP molecules; thus, the reaction rate provides a measure of the total accessible antigen binding sites presented by the immobilized antibody. First, note that the native antibody control did not produce a detectable signal. This confirms that biotin is required to immobilize the antibody onto the plate and that HRP does not nonspecifically bind to the streptavidin plate. As the concentration of the biotinylated antibody used to functionalize the streptavidin wells increased, the number of HRP molecules captured increased, until reaching a maximum binding capacity that indicates that the well was completely coated with a monolayer of antibody (Figure 4). A lower solution concentration of random biotinylated antibody (e.g., 10 nM) was required to fully coat the streptavidin well compared to the site-specific biotinylated antibody (e.g., ∼50–100 nM). An IgG molecule has molecular dimensions of ∼4.5 × 8.5 × 14 nm;^3^ therefore, its molecular footprint varies based on immobilized orientation.^52^ It is expected that the average molecular footprint is smaller and packing density is greater for the antibody adsorbed in a controlled orientation via the biotinylated Fc region compared to the randomly biotinylated antibody; thus, a greater concentration of site-specific biotinylated antibody is required to realize monolayer coverage in the well. As anticipated, the data in Figure 4 also reveal that a monolayer of the oriented antibody yields a greater antigen binding capacity than that of the randomly oriented antibody. This is likely due to greater surface density, more accessible binding sites, or a combination of these effects that is achieved with a site-specific biotinylated antibody. It is noted that crowding effects related to densely packed immobilized antibodies that negatively impact antigen binding are not observed in this system (i.e., HRP capture does not decrease at excessively high antibody concentrations) and may be due to streptavidin coverage.^49−51^ The commercial streptavidin-coated plates selected for this work are designed for immobilizing capture antibodies for immunoassay applications. It is probable that the distribution of immobilized streptavidin was optimized specifically to prevent overcrowding. By contrast, a high-capacity streptavidin plate is also available but not recommended for use in an immunoassay by the manufacturer, and while no rationale is provided, the high-capacity plate likely leads to overcrowding effects and diminished immunoassay performance.

**Figure 5:**
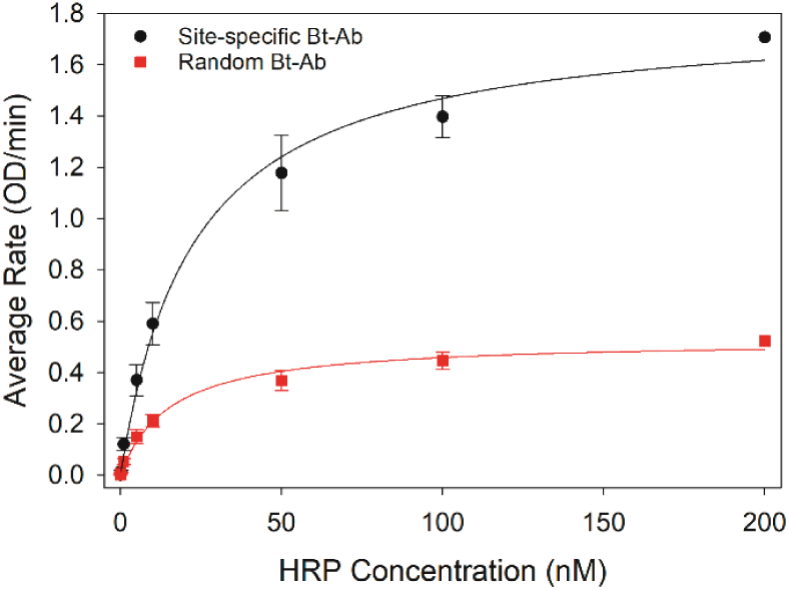
Dependence of a functional assay to bind HRP on the antibody immobilization strategy. Site-specific and random biotinylated anti-HRP antibodies were immobilized on a streptavidin-coated 96-well plate. The dose-dependent binding of HRP to the immobilized antibodies was determined from the rate of HRP-catalyzed ABTS oxidation. Data points and error bars represent the average values and standard deviations determined from three independent experiments.

An immunoassay for the detection of HRP was performed using both random and site-specific biotinylated antibodies as capture antibodies to compare analytical performance. Each antibody was adsorbed onto a streptavidin-coated multiwell plate at a constant concentration of 100 nM. Guided by the results presented in Figure 4, this antibody concentration is sufficient to fully saturate each well of the plate with a biotinylated antibody and maximize the number of antigen-binding sites. Solutions of HRP with varying concentrations were added to the wells for binding to the capture antibody. The quantity of captured antigen was determined from the rate of the HRP–ABTS enzymatic assay (Figure S6). Both capture antibodies generated a dose-dependent response, where initially the signal increased as the antigen concentration increased before plateauing at a maximum signal when the capture antibodies were saturated with antigens (Figure 5). Notably, for each standard solution of HRP, the oriented capture antibody (e.g., site-specific biotinylated anti-HRP antibody) captured more HRP molecules than the randomly oriented. The greater number of binding sites presented by the oriented antibody shifts equilibrium to the bound state, resulting in more captured HRP molecules and greater signal for any given antigen concentration compared to the randomly immobilized antibody. The ratio of signals for the site-specific to the randomly biotinylated capture antibodies was calculated and is presented in Table S1. The oriented capture antibody provides an ∼2× greater signal than the randomly oriented antibody at low antigen concentrations, where binding sites are in excess of antigen. The ratio increases to a maximum of ∼3× at high antigen concentrations, where binding sites become limited. Overall, the site-specific biotinylated antibody improves the analytical sensitivity 2–3× compared to the randomly biotinylated antibody (Table S2). The detection limit for the immunoassays using site-specific and randomly biotinylated antibodies was 9 and 29 pM, respectively, determined as the antigen concentration that generates a signal greater than the blank signal plus three times the standard deviation of the blank signal (Table S2).

Conventional immunoassays (e.g., ELISAs) are most commonly prepared using a native antibody adsorbed onto a polystyrene plate via hydrophilic interactions to form the capture substrate. This approach results in randomly oriented capture antibodies, and it is conceivable to expect an analytical performance similar to that achieved by the randomly biotinylated antibody immobilized on the streptavidin-coated plate. To confirm this supposition and to establish that controlled orientation of the capture antibody through site-specific biotinylation is advantageous, a native anti-HRP antibody was adsorbed on a high-binding polystyrene 96-well plate at saturating concentrations. An immunoassay for this conventional capture substrate was performed for HRP detection, and the dose–response curve is provided in Figure S7. An expected ligand binding curve was observed, and as anticipated, the sensitivity and detection limit were substantially worse than those of the immunoassay performed with the site-specific biotinylated antibody on a streptavidin plate (Table S2). The analytical performance of the assay using this physically adsorbed antibody is slightly worse than the randomly biotinylated antibody on the streptavidin plate. Several factors may contribute to the diminished performance of the nonspecifically adsorbed antibody compared to the randomly biotinylated antibody, even though both were randomly oriented. First, weaker adsorption of the native antibody to the polystyrene plate compared to the high-affinity biotin–streptavidin immobilization strategy may lead to antibody desorption. Second, loss of function because of adsorption-induced denaturation of the antibody may contribute to reduced antigen binding. Third, it is notable that the randomly biotinylated antibody has four PEG units spacing the antibody from the solid substrate, whereas the nonspecifically adsorbed antibody in the ELISA is not displaced from the substrate. The increased spacing provided by the PEG_4_ linker may reduce steric hindrance and increase accessibility to the Fab.^53,54^

Protein A and protein G are readily accessible and easily implemented approaches for the oriented immobilization of IgG antibodies. As a benchmark for comparison to the mTG-mediated site-specific biotinylation and immobilization of the anti-HRP antibody, a streptavidin-coated 96-well plate was functionalized with biotinylated protein G. Subsequently, an anti-HRP antibody was bound via the protein G layer to form an oriented capture antibody layer, and this substrate was evaluated for HRP capture (Figure S7 and Table S2). It was hypothesized that the analytical performance achieved by protein G-mediated immobilization would be similar to that of the mTG site-specific biotinylated antibody, assuming that a robust antibody layer was formed. The expected concentration-dependent ligand binding curve was observed; however, the protein G-immobilized anti-HRP antibody resulted in substantially worse sensitivity and limit of detection (199 pM). As previously discussed, the affinity of Fc-binding proteins varies greatly across different antibody types (e.g., host species and subtypes). Thus, the poor analytical performance is attributed to the instability of the protein G–antibody binding for this system and clearly demonstrates the advantage of mTG in facilitating oriented immobilization.

## Conclusions

We developed a new strategy for oriented immobilization of antibodies using mTG to facilitate surface attachment through the Fc region with site-specificity. mTG catalyzed the covalent coupling of the amine group on an NH_2_-PEG_4_-biotin to Q295 on the Fc heavy chain with high efficiency and specificity. Chemo–enzymatic cross-coupling efficiency was optimized by deglycosylation of the antibody to yield 1.9 biotins per antibody with a theoretical maximum of 2 biotins per antibody. Western blot analysis confirmed biotinylation of only the heavy chains, supporting site-directed conjugation via mTG. The practical application of this strategy to fabricate a capture substrate with oriented antibodies was demonstrated by a functional assay. This novel approach realized a 3-fold improvement in analytical detection benchmarked to an assay performed with randomly oriented capture antibodies immobilized on the biosensor via biotinylated antibodies using conventional conjugation chemistry. Furthermore, our results suggest that improvements in analytical performance may be possible through optimization of the PEG spacer length. Comparative analysis of the ELISA using a directly adsorbed native antibody (0-spacer) and the randomly biotinylated antibody (PEG_4_-spacer) indicates that accessibility of the Fab for the immobilized antibody is likely impacted by the spacer length. Future efforts will explore this opportunity to minimize the steric hindrance imposed by the support substrate to realize the full analytical potential of this immobilization method.

This strategy to properly orient immobilized antibodies provides many advantages compared with other approaches. Importantly, this method potentially offers tremendous flexibility to covalently install a wide variety of bioaffinity elements (e.g., biotin, histidine tag, and SpyTag) or chemical linking moieties (e.g., thiol and azide) for oriented immobilization on an appropriately matched surface. Moreover, this site-directed conjugation to the Fc region is expected to be universally applicable to most IgGs, independent of host species or subtype, overcoming a major challenge associated with oriented immobilization approaches relying on established or novel Fc-binding ligands.

## Supporting Information Available

UV–visible spectra of biotin quantitation via HABA; SDS-PAGE of biotinylated antibodies; the sequence alignment of rat and human IgG heavy chains; SPR sensograms of antibody–antigen binding; kinetics curves for the HRP-catalyzed reaction; conventional ELISA and protein G immobilized antibodies for HRP capture; the table comparing assay signals for the random and site-specific biotinylated capture antibody; and the table comparing the assay analytical sensitivity and detection limit (PDF)
