# Supplementary material for: Oriented Surface Immobilization of Antibodies Using Enzyme-Mediated Site-Specific Biotinylation for Enhanced Antigen-Binding Capacity
Source: Langmuir. 2025 Apr 20;41(16):10576–85. doi: 10.1021/acs.langmuir.5c00656 (PMC12044683; doi:10.1021/acs.langmuir.5c00656)
Supplement: Supplementary file 1 — la5c00656_si_001.pdf [file la5c00656_si_001.pdf]

SUPPORTING INFORMATION FOR:

Oriented surface immobilization of antibody using enzyme-mediated site-specific biotinylation for enhanced antigen binding capacity

*Emily Beitello, Kwame Osei, Trent Kobulnicky, Faith Breausche, Jon A. Friesen, and Jeremy D. Driskell**

Department of Chemistry, Illinois State University, Normal, IL USA 61790

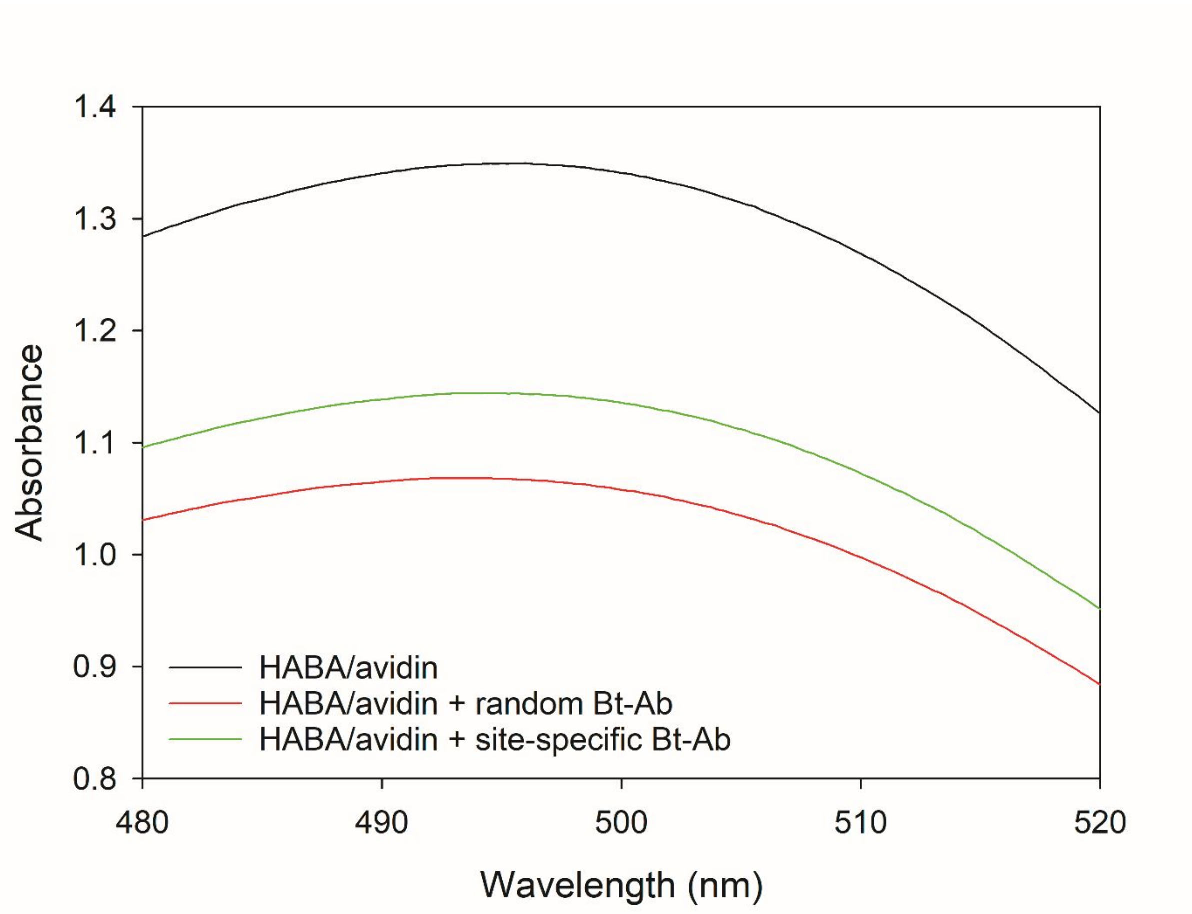

Figure S1. Representative UV-vis spectra for Bt quantitation via HABA. Spectra were recorded from 480-520 nm for the HABA/avidin complex before addition of biotinylated antibody and after the addition of random or site-specific biotinylated antibody. The decrease in absorbance at 500 nm correlates to the number of biotin elements conjugated to the antibody.

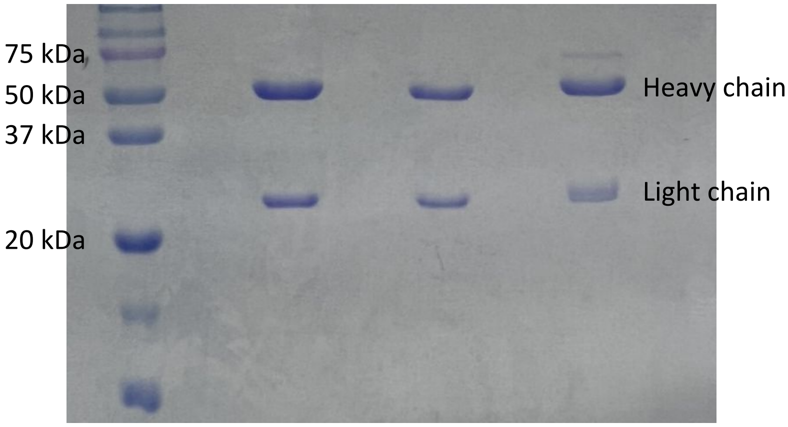

Figure S2. SDS-PAGE of native, site-specific, and random biotinylated antibody under reducing conditions and stained with Coomassie blue. Lanes (left to right): molecular weight ladder, *empty*, native rat anti-HRP antibody, *empty*, site-specific biotinylated rat anti-HRP antibody, *empty*, random biotinylated rat anti-HRP antibody.

```
                                                                                   Q295    N297
Rat IgG1       SVFLFPPKPKDTLMISRTPEVTCVVVDVSHEDPQVKFNWYVDGVQVHNAKTKPREQQYNSTYRVVSV
Human IgG1     DVFLFPPKPKDTLMISRTPEVTCVVVDVSHEDPEVKFNWYVDGVEVHNAKTKPREEQYNSTYRVVSV
```

Figure S3. Multiple sequence alignment for the heavy chain of rat and human IgGs showing the highly conserved Q295 and N297. This multiple sequence alignment was generated using Kalign software.

A **B**

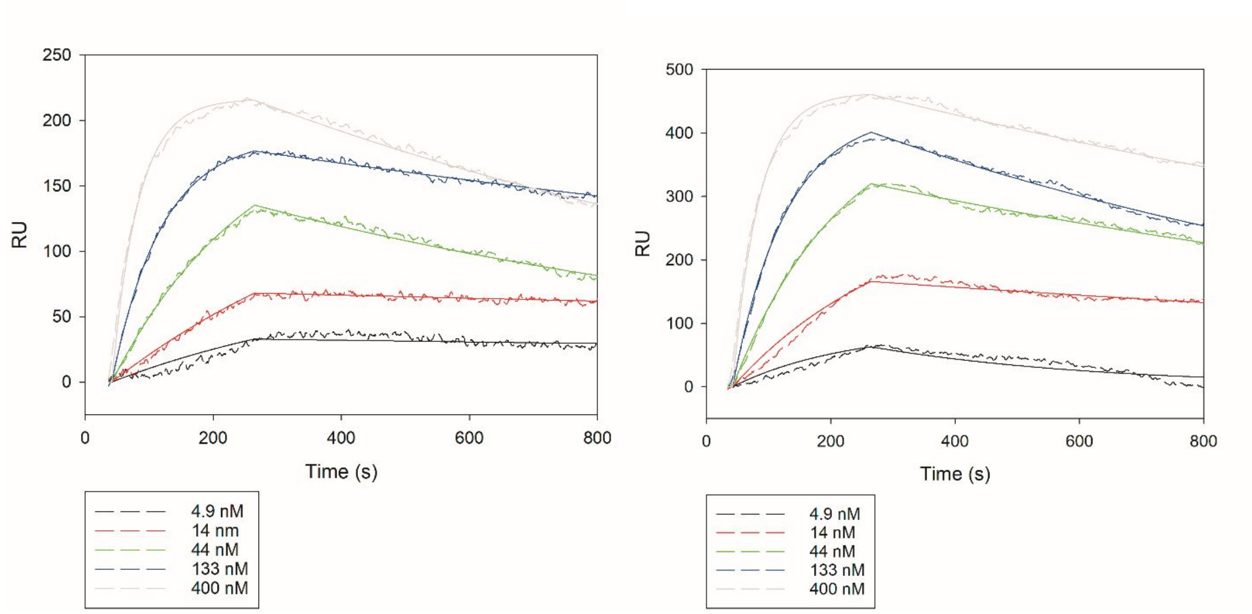

Figure S4. SPR sensograms for the binding of HRP at 4.9, 14, 44, 133, and 400 nM to immobilized anti-HRP antibody, followed by rinsing with PBS-T to monitor dissosication. The streptavidin coated SPR sensor was functionalized with random (A) and site-specific (B) biotinylated anti-HRP antibody. Binding model fits (solid lines) are overlaid on the experimental signal (dashed lines).

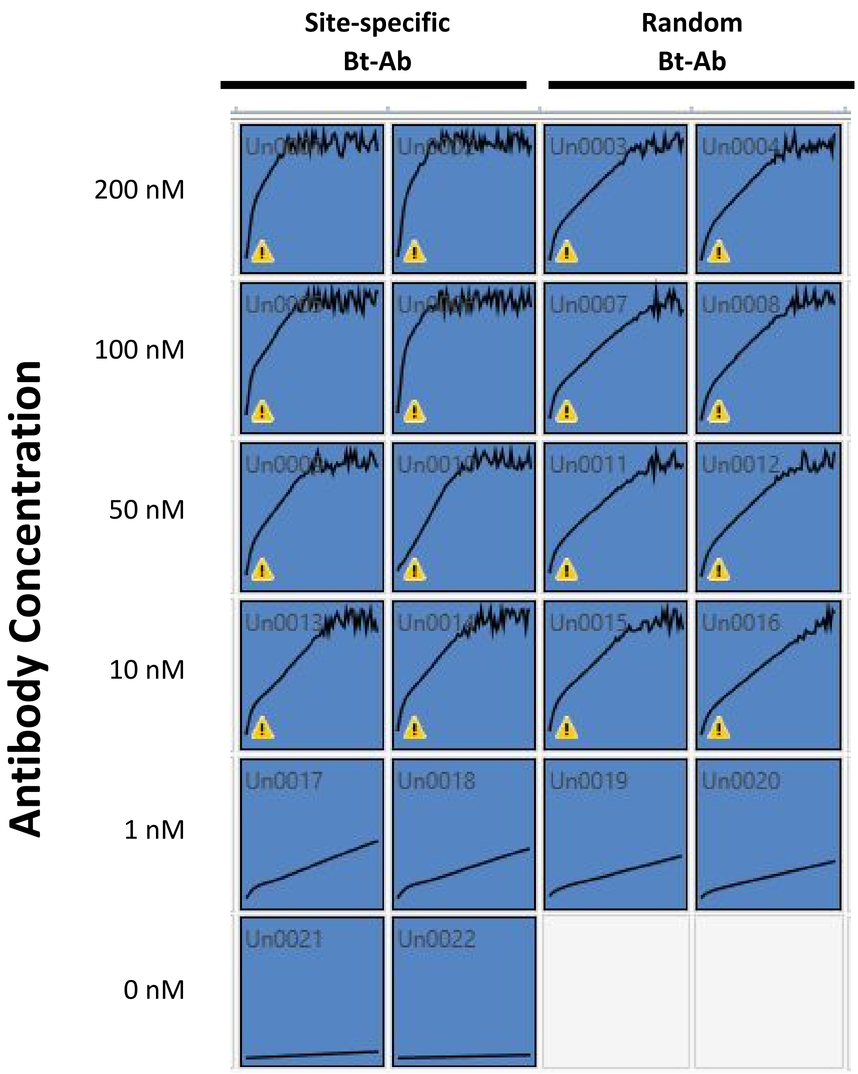

Figure S5. Absorbance versus time for HRP-catalyzed oxidation of ABTS. Varying concentrations of site-specific and random biotinylated anti-HRP antibody was used to functionalize streptavidin-coated wells and accessible binding sites were saturated with HRP to determine antibody concentration required to fully coat wells. Each antibody concentration and antibody immobilization strategy were plated in duplicate. Reaction rate was calculated as the averages slope of absorbance versus time for the linear portion of the kinetics curve.

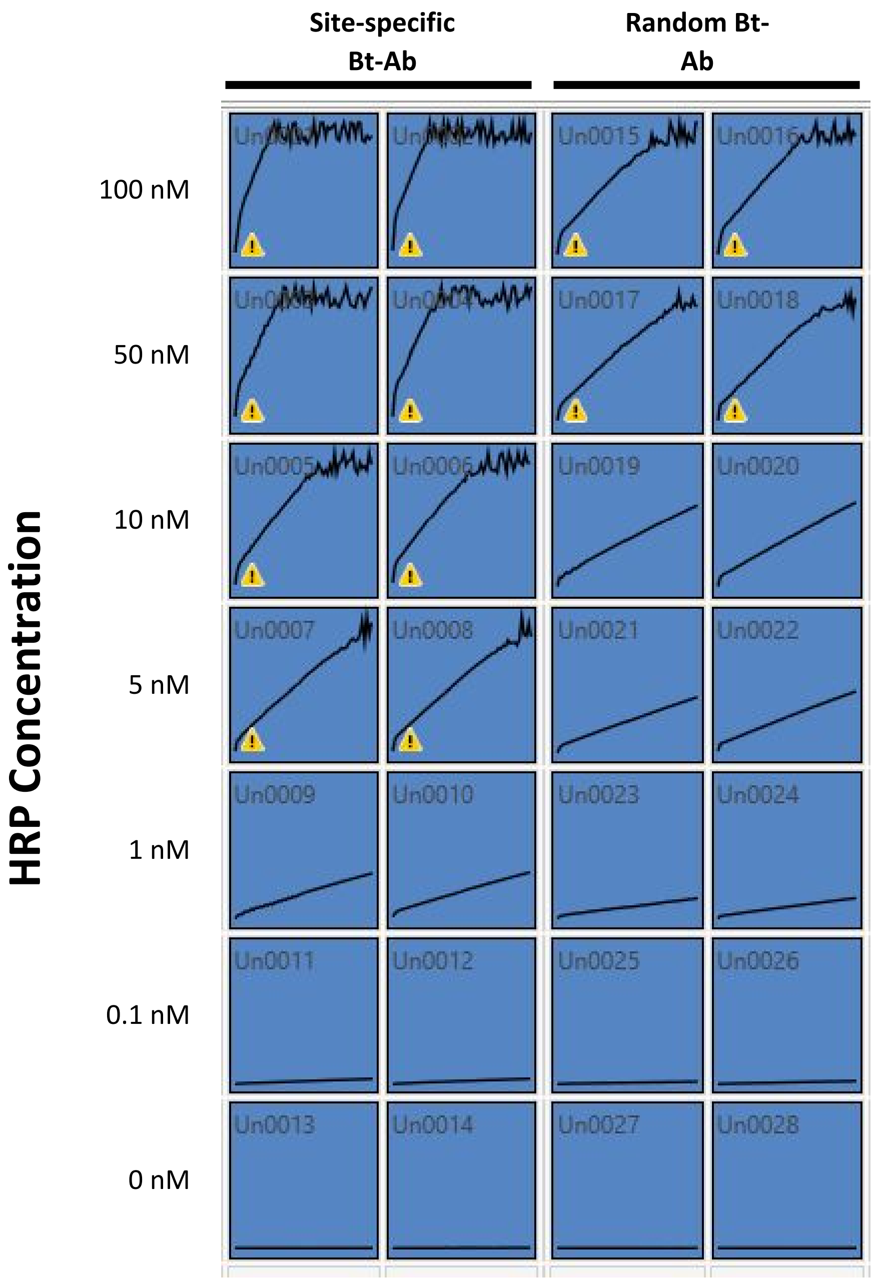

Figure S6. Absorbance versus time for HRP-catalyzed oxidation of ABTS. Varying concentrations of HRP were captured by site-specific and random biotinylated anti-HRP antibody immobilized on a streptavidin-coated well at saturating levels. Each HRP concentration and antibody immobilization strategy were plated in duplicate. Reaction rate was calculated as the averages slope of absorbance versus time for the linear portion of the kinetics curve.

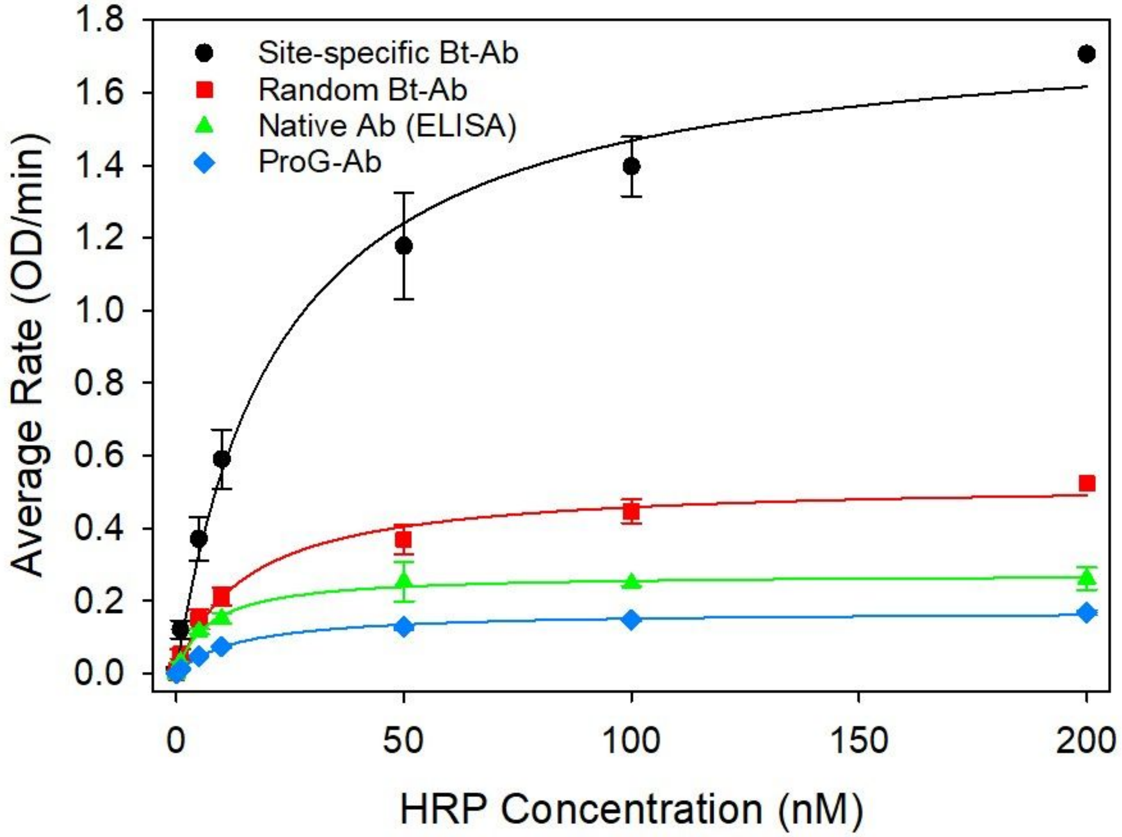

Figure S7. Dependence of a functional assay to bind HRP on the antibody immobilization strategy. Site-specific and random biotinylated anti-HRP antibody were immobilized on a streptavidin coated 96-well plate. Native (non-biotinylated) anti-HRP antibody was adsorbed onto an ELISA plate. Biotinylated protein G was immobilized onto a streptavidin coated 96-well plate, followed by the oriented capture of native anti-HRP antibody. The dose-dependent binding of HRP to the immobilized antibodies was determined from the rate of HRP-catalyzed ABTS oxidation.

Table S1. The rate of HRP-catalyzed ABTS oxidation measured for HRP binding assays conducted by immobilization of site-specific and random biotinylated antibodies.

HRP Concentration (nM)	Site-specific Bt-Ab Rate (OD/min)	Random Bt-Ab Rate (OD/min)	Ratio (Site-specific:Random)
0	0.001	0.001	-
0.1	0.014	0.007	2.15
1	0.121	0.053	2.27
5	0.370	0.149	2.48
10	0.590	0.212	2.78
50	1.178	0.368	3.20
100	1.397	0.447	3.13
200	1.707	0.523	3.27
**Average Ratio (≥50 nM)**			**3.20**

Table S2. Sensitivity and detection limit for HRP immunoassay performed using site-specific Bt-Ab, random Bt-Ab, native Ab (ELISA), and protein G immobilized Ab. Using the linear portion of the calibration curve in Figures 5 and S7, the sensitivity is defined as the slope and detection limit is defined as the antigen concentration that generates a signal greater than the blank signal plus three times the standard deviation of the blank signal.

**Capture Ab**	**Detection Limit (pM)**	**Sensitivity ((OD/min)/nM)**
Site-specific Bt-Ab	9.3	0.1196
Random Bt-Ab	29	0.0521
Native Ab (ELISA)	88	0.0319
Protein G-Ab	199	0.0121
